# Non contiguous-finished genome sequence and description of *Peptoniphilus grossensis* sp. nov.

**DOI:** 10.4056/sigs.3076460

**Published:** 2012-12-19

**Authors:** Ajay Kumar Mishra, Perrine Hugon, Catherine Robert, Didier Raoult, Pierre-Edouard Fournier

**Affiliations:** Unité de Recherche sur les Maladies Infectieuses et Tropicales Emergentes, UMR CNRS 7278

**Keywords:** *Peptoniphilus grossensis*, genome

## Abstract

*Peptoniphilus grossensis* strain ph5^T^ sp. nov., is the type strain of *Peptoniphilus grossensis* sp. nov., a new species within the *Peptoniphilus* genus. This strain, whose genome is described here, was isolated from the fecal flora of a 26-year-old woman suffering from morbid obesity. *P. grossensis* strain ph5 is a Gram-positive obligate anaerobic coccus. Here we describe the features of this organism, together with the complete genome sequence and annotation. The 2,101,866-bp long genome (1 chromosome but no plasmid) exhibits a G+C content of 33.9% and contains 2,041 protein-coding and 29 RNA genes, including 3 rRNA genes.

## Introduction

*Peptoniphilus grossensis* strain ph5^T^ (= CSUR P184 = DSM 25475), is the type strain of *Peptoniphilus grossensis* sp. nov. This bacterium is a Gram-positive, spore-forming, indole positive, anaerobic coccoid bacterium that was isolated from the stool of a 26-year-old woman suffering from morbid obesity.

Since 1995 and the sequencing of the first bacterial genome, that of *Haemophilus influenzae*, more than 3,000 bacterial genomes have been sequenced [1]. This was permitted by technical improvements as well as increased interest in having access to the complete genetic information encoded by bacteria. At the same time, biological tools for defining new bacterial species have not evolved, and DNA-DNA hybridization is still considered the gold standard [2] despite its drawbacks and the taxonomic revolution that has resulted from the comparison of 16S rDNA sequences [3]. In this manuscript, we propose and describe a new *Peptoniphilus* species using genomic and phenotypic information [4] to.

Gram-positive anaerobic cocci (GPAC) are part of the commensal flora of humans and animals, and are also commonly associated with a variety of human infections [5,6]. Extensive taxonomic changes have occurred in this group of bacteria, especially in clinically-important genera such as *Finegoldia*, *Micromonas*, and *Peptostreptococcus* [7]. The genus *Peptostreptococcus* was divided into three genera: *Peptoniphilus* (Ezaki *et al*., 2001), *Anaerococcus* (Ezaki *et al*., 2001) and *Gallicola* (Ezaki *et al*., 2001). The genus *Peptoniphilus* includes the following butyrate-producing, non-saccharolytic species that use peptone and amino acids as major energy sources: *P. asaccharolyticus*, *P. gorbachii*, *P. harei*, *P. indolicus*, *P. ivorii*, *P. lacrimalis* [7], *P. olsenii* [8] and *P. methioninivorax* [9].

Members of the genus *Peptoniphilus* have mostly been isolated from various human clinical specimens such as vaginal discharges, ovarian, peritoneal, sacral and lacrymal gland abscesses [7]. In addition, *P. indolicus* causes summer mastitis in cattle [7].

Here we present a summary classification and a set of features for *P. grossensis* sp. nov. strain ph5^T^ (= CSUR P184 = DSM 25475), together with the description of the complete genomic sequencing and annotation. These characteristics support the circumscription of the species *P. grossensis*.

## Classification and features

**Figure 1:**
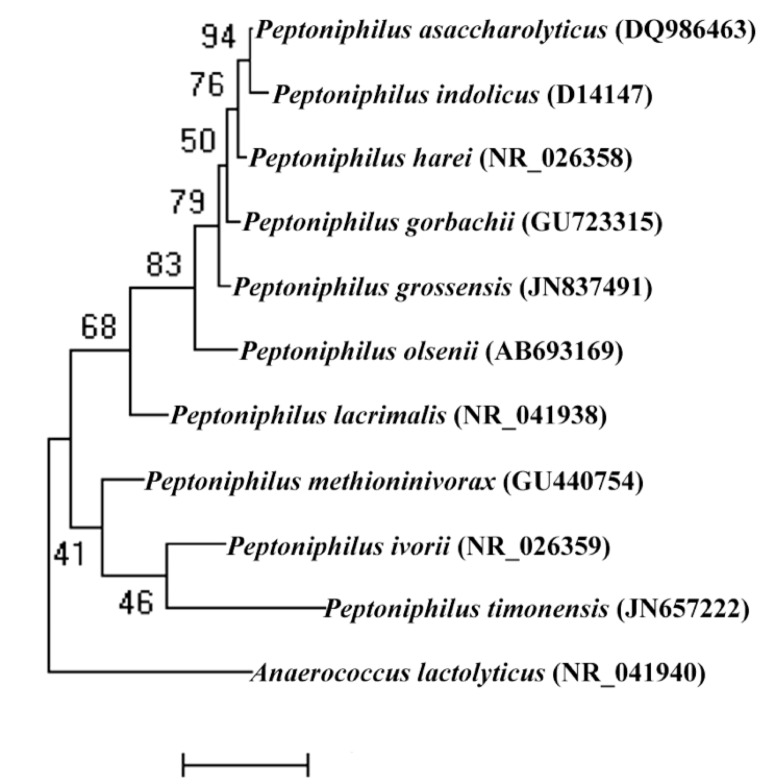
Phylogenetic tree highlighting the position of *Peptoniphilus grossensis* strain ph5^T^ relative to other type strains within the *Peptoniphilus* genus. GenBank accession numbers are indicated in parentheses. Sequences were aligned using CLUSTALW, and phylogenetic inferences obtained using the maximum-likelihood method within MEGA program. Numbers at the nodes are percentages of bootstrap values obtained by repeating the analysis 500 times to generate a majority consensus tree. *Anaerococcus lactolyticus* was used as an outgroup. The scale bar represents a 2% nucleotide sequence divergence.

A stool sample was collected from a 26-year-old woman living in Marseille, France, who suffered from morbid obesity: BMI=48.2 (118.8 kg, 1.57 meter). At the time of stool sample collection, she was not a drug-user and was not on a diet. The patient gave an informed and signed consent, and the agreement of local ethics committee of the IFR48 (Marseille, France) were obtained under agreement 11-017. The fecal specimen was preserved at -80°C after collection. Strain PH5^T^ (Table 1) was isolated in 2011 by anaerobic cultivation on 5% sheep blood-enriched Columbia agar (BioMerieux, Marcy l’Etoile, France) after 26 days of preincubation of the stool sample in rumen and ship blood bottle culture. This strain exhibited a 96.7% nucleotide sequence similarity with *P. harei* and occupied an intermediate phylogenetic position between *P. gorbachii* and *P. olsenii* (Figure 1). Although sequence similarity of the 16S operon is not uniform across taxa, this value was lower than the 98.7% 16S rRNA gene sequence threshold recommended by Stackebrandt and Ebers to delineate a new species without carrying out DNA-DNA hybridization [3].

**Table 1:** Classification and general features of *Peptoniphilus grossensis* strain ph5^T^ according to the MIGS recommendations [10]

**MIGS ID**	**Property**	**Term**	**Evidence code^a^**
	Current classification	Domain *Bacteria*	TAS [11]
		Phylum *Firmicutes*	TAS [12-14]
		Class *Clostridia*	TAS [15,16]
		Order *Clostridiales*	TAS [17,18]
		Family *Clostridiales* family XI *Incertae sedis*	NAS
		Genus *Peptoniphilus*	TAS [7]
		Species *Peptoniphilus grossensis*	IDA
		Type strain: ph5	IDA
	Gram stain	Positive	IDA
	Cell shape	Coccoid	IDA
	Motility	Nonmotile	IDA
	Sporulation	Sporulating	IDA
	Temperature range	Mesophile	IDA
	Optimum temperature	37°C	IDA
MIGS-6.3	Salinity	growth in BHI medium + 5% NaCl	IDA
MIGS-22	Oxygen requirement	Anaerobic	IDA
	Carbon source	Unknown	
	Energy source	Peptones	NAS
MIGS-6	Habitat	human gut	IDA
MIGS-15	Biotic relationship	free living	IDA
MIGS-14	Pathogenicity	Unknown	
	Biosafety level	2	
	Isolation	human feces	
MIGS-4	Geographic location	France	IDA
MIGS-5	Sample collection time	January 2011	IDA
MIGS-4.1	Latitude	43.296482	IDA
MIGS-4.2	Longitude	5.36978	IDA
MIGS-4.3	Depth	Surface	IDA
MIGS-4.4	Altitude	0 m above sea level	IDA

Evidence codes - IDA: Inferred from Direct Assay; TAS: Traceable Author Statement (i.e., a direct report exists in the literature); NAS: Non-traceable Author Statement (i.e., not directly observed for the living, isolated sample, but based on a generally accepted property for the species, or anecdotal evidence). These evidence codes are from the Gene Ontology project [19]. If the evidence is IDA, then the property was directly observed for a live isolate by one of the authors or an expert mentioned in the acknowledgements.

**Figure 2:**
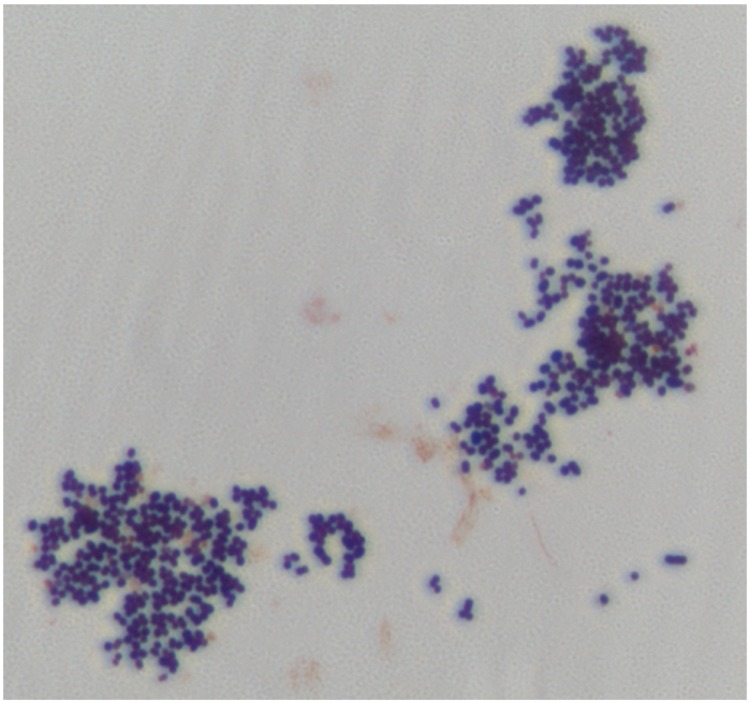
Gram staining of *P. grossensis* strain ph5^T^

**Figure 3:**
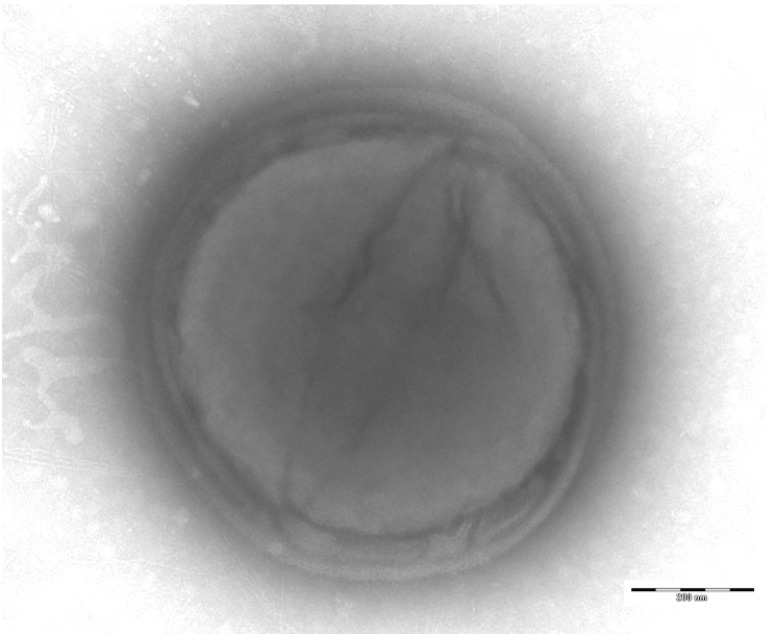
Transmission electron microscopy of *P. grossensis* strain ph5^T^, using a Morgani 268D (Philips) at an operating voltage of 60kV. The scale bar represents 900 nm.

Different growth temperatures (25, 30, 37, 45°C) were tested; no growth occurred at 25°C, 30°C or 45°C. Growth only occurred at 37°C. Colonies were 2 mm in diameter on blood-enriched Columbia agar and Brain Heart Infusion (BHI) agar. Growth of the strain was tested under anaerobic and microaerophilic conditions using GENbag anaer and GENbag microaer systems, respectively (BioMérieux), and in the presence of air, with or without 5% CO_2_. Growth was achieved only anaerobically. Gram staining showed Gram-positive cocci able to form spores (Figure 2). The motility test was negative. Cells grown on agar had a mean diameter of 1.2 µm by electron microscopy and were mostly grouped in pairs, short chains or small clumps (Figure 3).

Strain ph5 exhibited neither catalase nor oxidase activities but indole production was observed. Using an API Rapid ID 32A strip (BioMerieux), a positive reaction was observed for Mannose fermentation, arginine arylamidase, tyrosine arylamidase, histidine arylamidase and leucine arylamidase. Strain ph5 was susceptible to penicillin G, amoxicillin, ceftriaxon, cefalexin, imipenem fosfomycin, erythromycin, doxycyclin, rifampin, vancomycin and metronidazole, but resistant to ciprofloxacin and cotrimoxazole.

**Figure 4:**
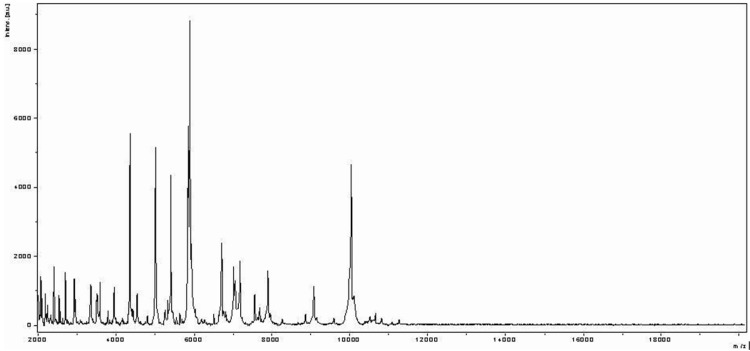
Reference mass spectrum from *P. grossensis* strain ph5^T^. Spectra from 12 individual colonies were compared and a reference spectrum was generated.

Matrix-assisted laser-desorption/ionization time-of-flight (MALDI-TOF) MS protein analysis was carried out as previously described [20]. Briefly, a pipette tip was used to pick one isolated bacterial colony from a culture agar plate and spread it as a thin film on a MTP 384 MALDI-TOF target plate (Bruker Daltonics, Germany). Twelve distinct deposits were done for strain ph5 from twelve isolated colonies. Each smear was overlaid with 2µL of matrix solution (saturated solution of alpha-cyano-4-hydroxycinnamic acid) in 50% acetonitrile, 2.5% tri-fluoracetic acid, and allowed to dry for five minutes. Measurements were performed with a Microflex spectrometer (Bruker). Spectra were recorded in the positive linear mode for the mass range of 2,000 to 20,000 Da (parameter settings: ion source 1 (ISI), 20kV; IS2, 18.5 kV; lens, 7 kV). A spectrum was obtained after 675 shots at a variable laser power. The time of acquisition was between 30 seconds and 1 minute per spot. The twelve ph5 spectra were imported into the MALDI Bio Typer software (version 2.0, Bruker) and analyzed by standard pattern matching (with default parameter settings) against the main spectra of 3,769 bacteria, including spectra from 8 validated *Peptoniphilus* species used as reference data, in the Bio Typer database (updated March 15^th^, 2012). The method of identification includes the m/z from 3,000 to 15,000 Da. For every spectrum, 100 peaks at most were taken into account and compared with the spectra in database. A score enabled the presumptive identification and discrimination of the tested species from those in a database: a score ≥ 2 with a validated species enabled the identification at the species level; a score ≥ 1.7 but < 2 enabled the identification at the genus level; and a score < 1.7 did not enable any identification. For strain ph5, the obtained score was 1.3, thus suggesting that our isolate was not a member of a known species. We incremented our database with the spectrum from strain ph5 (Figure 4).

## Genome sequencing information

### Genome project history

The organism was selected for sequencing on the basis of its phylogenetic position and 16S rRNA similarity to other members of the genus *Peptoniphilus*. To date, the genomes from only tree validated *Peptoniphilus* species have been sequenced. This was the first genome of *Peptoniphilus grossensis* sp. nov. A summary of the project information is shown in Table 2. The Genbank accession number is CAGX00000000 and consists of 77 contigs. Table 2 shows the project information and its association with MIGS version 2.0 compliance.

**Table 2:** Project information

**MIGS ID**	**Property**	**Term**
MIGS-31	Finishing quality	High-quality draft
MIGS-28	Libraries used	One 454 paired end 3-kb library
MIGS-29	Sequencing platforms	454 GS FLX Titanium
MIGS-31.2	Sequencing	26.78×
MIGS-30	Assemblers	Newbler version 2.5.3
MIGS-32	Gene calling method	Prodigal
	INSDC ID	PRJEB48
	Genbank ID	CAGX00000000
	Genbank Date of Release	May 30, 2012
	Gold ID	Gi13722
MIGS-13	Project relevance	Study of the human gut microbiome

### Growth conditions and DNA isolation

*P. grossensis* strain ph5^T^ (= CSUR P184 = DSM 25475), was grown on blood agar medium at 37°C. Six petri dishes were spread and resuspended in 6×100µl of G2 buffer (EZ1 DNA Tissue kit, Qiagen). A first mechanical lysis was performed by glass powder on the Fastprep-24 device (Sample Preparation system) from MP Biomedicals, USA) using 40 seconds cycles. DNA was then treated with 2.5 µg/µL lysozyme (30 minutes at 37°C) and extracted through the BioRobot EZ 1 Advanced XL (Qiagen). The DNA was then concentrated and purified on a Qiamp kit (Qiagen). The yield and the concentration was measured by the Quant-it Picogreen kit (Invitrogen) on a Genios_Tecan fluorometer at 62 ng/µl.

### Genome sequencing and assembly

DNA (5µg) was mechanically fragmented on the Hydroshear device (Digilab, Holliston, MA, USA) with an enrichment size of 3-4kb. The DNA fragmentation was visualized using an Agilent 2100 BioAnalyzer on a DNA labchip 7500 to yield an optimal size of 3.16 kb. The library was constructed according to the 454_Titanium paired-end protocol and manufacturer. Circularization and nebulization were performed and generated a pattern with an optimum at 628 bp. After PCR amplification through 15 cycles followed by double size selection, the single stranded paired end library was then quantified on the Quant-it Ribogreen kit (Invitrogen) on the Genios_Tecan fluorometer at 34pg/µL. The library concentration equivalence was calculated as 9.93E+08 molecules/µL. The library was held at -20°C until use.

The shotgun library was clonally amplified with 0.5 and 1 cpb in 2 emPCR reactions per condition with the GS Titanium SV emPCR Kit (Lib-L) v2. The yields of the emPCR at 0.5cpb and 1 cpb were of 9.63% and 22.35%, respectively. A total of 790,000 beads for a ¼ region and 790,000 beads for a 1/8 region were loaded on the GS Titanium PicoTiterPlates (PTP Kit 70×75) and sequenced with the GS Titanium Sequencing Kit XLR70.

The runs were performed overnight and then analyzed on the cluster through the gsRunBrowser and gsAssembler_Roche. The global 176,029 passed filter sequences generated 56.24 Mb with a length average of 319 bp. These sequences were assembled using the Newbler software from Roche with 90% identity and 40 bp as overlap. Seventy-seven large contigs (>1500bp) were obtained, for a genome size of 2.1Mb which corresponds to a coverage of 26.78× genome equivalent.

### Genome annotation

Open Reading Frames (ORFs) were predicted using Prodigal [21] with default parameters but the predicted ORFs were excluded if they were spanning a sequencing gap region. The predicted bacterial protein sequences were searched against the GenBank database [22] and the Clusters of Orthologous Groups (COG) databases using BLASTP. The tRNAScanSE tool [23] was used to find tRNA genes, whereas ribosomal RNAs were found by using RNAmmer [24] and BLASTn against the GenBank database. Lipoprotein signal peptides and numbers of transmembrane helices were predicted using SignalP [25] and TMHMM [26] respectively. ORFans were identified if their BLASTP *E*-value was lower than 1e-3 for alignment length greater than 80 amino acids. If alignment lengths were smaller than 80 amino acids, we used an *E*-value of 1e-05. Such parameter thresholds have already been used in previous works to define ORFans. To estimate the mean level of nucleotide sequence similarity at the genome level between *Peptoniphilus* species, we compared the ORFs only using BLASTN and the following parameters: a query coverage of ≥ 70% and a minimum nucleotide length of 100 bp. Artemis [27] were used for data management and DNA Plotter [28] was used for visualization of genomic features. Mauve alignment tool was used for multiple genomic sequence alignment and visualization [29].

## Genome properties

**Figure 5:**
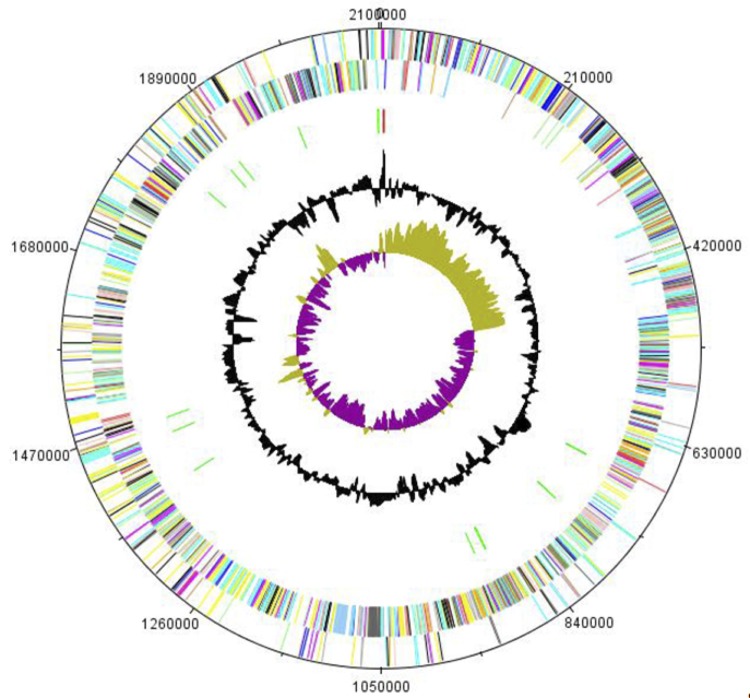
Graphical circular map of the chromosome. From the outside to the center: genes on the forward strand (colored by COG categories), genes on the reverse, RNA genes (rRNAs red, tRNAs green), G+C content, GC skew (purple negative values, olive positive values).

The genome of *P. grossensis* sp. nov. strain ph5^T^ is 2,101,866 bp long (1 chromosome, but no plasmid) with a 33.9% G + C content of (Figure 5 and Table 3). Of the 2,070 predicted genes, 2,041 were protein-coding genes, and 29 were RNAs. Three rRNA genes (one 16S rRNA, one 23S rRNA and one 5S rRNA) and 26 predicted tRNA genes were identified in the genome. A total of 1,439 genes (69.52%) were assigned a putative function. One hundred and fifty-five genes were identified as ORFans (7.6%). The remaining genes were annotated as hypothetical proteins. The properties and statistics of the genome are summarized in Table 3. The distribution of genes into COGs functional categories is presented in Table 4.

**Table 3:** Nucleotide content and gene count levels of the genome

**Attribute**	**Value**	**% of total^a^**
Genome size (bp)	2,101,866	
DNA coding region (bp)	1,919,775	91.34
DNA G+C content (bp)	712,533	33.9
Number of replicons	1	
Extrachromosomal elements	0	
Total genes	2,070	100
RNA genes	29	1.42
rRNA operons	1	
Protein-coding genes	2,041	98.59
Genes with function prediction	1,418	68.50
Genes assigned to COGs	1,439	69.52
Genes with peptide signals	128	6.18
Genes with transmembrane helices	542	26.18

^a^ The total is based on either the size of the genome in base pairs or the total number of protein coding genes in the annotated genome

**Table 4:** Number of genes associated with the 25 general COG functional categories

**Code**	**Value**	**%age**^a^	**Description**
J	137	6.71	Translation
A	0	0	RNA processing and modification
K	117	5.73	Transcription
L	130	6.36	Replication, recombination and repair
B	1	0.05	Chromatin structure and dynamics
D	20	0.98	Cell cycle control, mitosis and meiosis
Y	0	0	Nuclear structure
V	68	3.33	Defense mechanisms
T	60	2.93	Signal transduction mechanisms
M	63	3.09	Cell wall/membrane biogenesis
N	6	0.29	Cell motility
Z	0	0	Cytoskeleton
W	0	0	Extracellular structures
U	25	1.22	Intracellular trafficking and secretion
O	63	3.08	Posttranslational modification, protein turnover, chaperones
C	98	4.80	Energy production and conversion
G	47	2.30	Carbohydrate transport and metabolism
E	127	6.22	Amino acid transport and metabolism
F	57	2.79	Nucleotide transport and metabolism
H	54	2.64	Coenzyme transport and metabolism
I	38	1.86	Lipid transport and metabolism
P	90	4.40	Inorganic ion transport and metabolism
Q	20	0.97	Secondary metabolites biosynthesis, transport and catabolism
R	197	9.65	General function prediction only
S	143	7.0	Function unknown
-	602	29.49	Not in COGs

^a^ The total is based on the total number of protein coding genes in the annotated genome.

## Comparison with genomes from other *Peptoniphilus* species

The genomes from only three validated *Peptoniphilus* species are currently available. Here, we compared the genome sequence of *P. grossensis* strain ph5^T^ with those of *P. harei* strain ACS-146-V-Sch2b, *P. duerdenii* strain ATCC BAA-1640, *P. lacrimalis* strain 315-B, as well as *P. timonensis* strain JC401^T^ that we recently studied.

**Figure 6:**
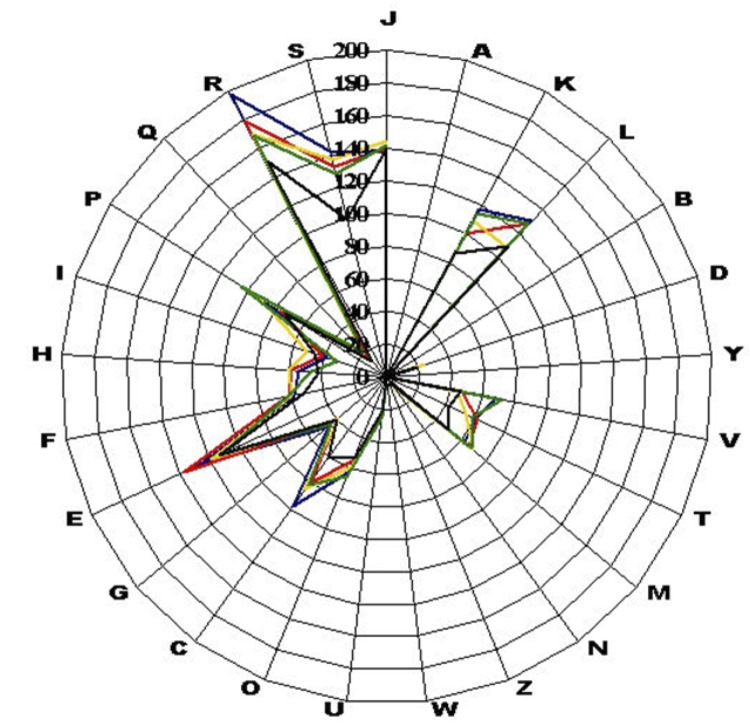
Compared distribution of predicted genes of *P. grossensis* (blue), *P. timonensis* (red), *P. harei* (yellow), *P. duerdenii* (green) and *P. lacrimalis* (black) into COG categories.

The draft genome sequence of *P. grossensis* strain ph5^T^ has a similar size to that of *P. duerdenii* (2.10 *vs* 2.12 Mb, respectively), but a larger size than *P. lacrimalis*, *P. harei* and *P. timonensis* (1.69, 1.83 and 1.75 Mb, respectively). The G+C content of *P. grossensis* is larger than *P. lacrimalis* and *P. timonensis* (33.9, 29.91 and 30.7%, respectively) and comparable to *P. duerdenii* and *P. harei* (34.24 and 34.44, respectively). The gene content of *P. grossensis* is larger than those of *P. duerdenii*, *P. lacrimalis*, *P. harei* and *P. timonensis* (2,041, 1,988, 1,636, 1,765 and 1,922, respectively). The ratio of genes per MB of *P. grossensis* is larger to those of *P. lacrimalis* and *P. harei* (986, 968 and 964, respectively) and smaller to those of *P. duerdenii* and *P. timonensis* (1,009 and 1,111, respectively). However, the distribution of genes into COG categories was highly similar in all four compared genomes (Figure 6). In addition, *P. grossensis* shares a mean 82.0% (range 70-99%), 85.8% (range 70.7-100%), 86.03 (range 70-100%) and 87.78% (range 70.8-100%) sequence similarity with *P. duerdenii*, *P. timonensis*, *P. harei* and *P. lacrimalis*, respectively, at the genome level. On the basis of phenotypic, phylogenetic and genomic analyses, we formally propose the creation of *Peptoniphilus grossensis* sp. nov. which includes strain ph5^T^. This bacterium has been found in Marseille, France.

## Description of *Peptoniphilus grossensis* sp. nov.

*Peptoniphilus grossensis* (gro.sen′sis. L. gen. masc. n. *grossensis*, of gros, the French adjective for fat, as the strain was isolated from an obese patient).

Colonies are 1 mm in diameter on blood-enriched Columbia agar and Brain Heart Infusion (BHI) agar. Cells are coccoid with a mean diameter of 1.2 μm, occurring mostly in pairs, short chains or small clumps. Growth is only achieved anaerobically. The optimal growth temperature is 37°C. Cells are Gram-positive, endospore-forming, and non-motile. Cells are negative for catalase and positive for indole production. Acid is produced from mannose. Positive reactions are observed for arginine arylamidase, tyrosine arylamidase, histidine arylamidase and leucine arylamidase. Cells are susceptible to penicillin G, amoxicillin, ceftriaxone, cefalexin, imipenem, fosfomycin, erythromycin, doxycyclin, rifampicin, vancomycin, metronidazole, but resistant to ciprofloxacin and cotrimoxazole. The G+C content of the genome is 33.9%. The genome and 16SrRNA sequences are deposited in GenBank under accession numbers CAGX00000000 and JN837491, respectively. The type strain ph5^T^ (= CSUR P184 = DSM 25475) was isolated from the fecal flora of an obese French patient.
